# The moderating role of HIV status in the association between gender identity and depression among sexual and gender minorities in Abuja, Nigeria

**DOI:** 10.1371/journal.pmen.0000590

**Published:** 2026-05-04

**Authors:** Ruxton Adebiyi, Chama John, Megan E. Mansfield, Andrew Mitchell, Jibreel Jumare, Rachel Sullivan Robinson, Typhanye Dyer, Rodman Turpin, Man Charurat, Sylvia Adebajo

**Affiliations:** 1 Institute of Human Virology, Nigeria; 2 Center for International Health, Education, and Biosecurity, Institute of Human Virology, University of Maryland School of Medicine, Baltimore, Maryland, United States of America; 3 Institute of Human Virology, University of Maryland School of Medicine, Baltimore, Maryland, United States of America; 4 Division of Global Health Sciences, Department of Epidemiology and Public Health, University of Maryland School of Medicine, Baltimore, Maryland, United States of America; 5 School of International Service, American University, Washington, District of Columbia, United States of America; 6 Department of Epidemiology and Biostatistics, University of Maryland School of Public Health, College Park, Maryland, United States of America; 7 Department of Global and Community Health, College of Public Health, George Mason University, Fairfax, Virginia, United States of America; National University of Singapore, SINGAPORE

## Abstract

In Nigeria, sexual and gender minorities (SGM) experience disproportionately high rates of depression, exacerbated by layered stigma related to gender identity and HIV status. However, little is known about how HIV status may modify the relationship between gender identity and depression in this context. We conducted a cross-sectional analysis using baseline data from 977 SGM participants recruited through respondent-driven sampling at an SGM-friendly clinic in Abuja, Nigeria (2023–2024). Depression was assessed using the Patient Health Questionnaire-9 (PHQ-9), with scores ≥10 indicating major depression symptomatology. We employed multivariable logistic regression to examine associations between gender identity, HIV status, and depression, including interaction effects. Overall, 18% of participants exhibited major depression symptomatology. Depressive symptoms were more common among transgender women (25%) and non-binary individuals (26%) than cisgender men (16%), and higher among persons living with HIV (PLHIV; 20%) compared to those without (15%). In adjusted models, both transgender women (aOR 2.05; 95% CI: 1.09–3.88) and non-binary individuals (aOR 2.38; 95% CI: 1.43–3.95) had higher odds of depression than cisgender men. Financial insecurity (aOR 5.78; 95% CI: 3.77–8.86) and employment (aOR 1.73; 95% CI: 1.18–2.52) were also independently associated with depression. Notably, the joint effect of non-cisgender identity and HIV was supra-additive: PLHIV who were non-binary had an aOR of 4.10 (95% CI: 2.16–7.77) for depression, with a relative excess risk due to interaction (RERI) of 2.96. HIV status modifies the association between gender identity and depression among SGM in Nigeria. These findings underscore the need for intersectional, stigma-informed mental health interventions and affirming care models that address the unique vulnerabilities faced by PLHIV with non-cisgender identities.

## Introduction

In Nigeria, addressing barriers to retention of vulnerable populations in Human Immunodeficiency Virus (HIV) prevention, treatment and care is key to reaching epidemic control. Such efforts require consideration of the quality of life of persons living with (PLHIV) and without HIV (PWoH). Evidence suggests that HIV status and other characteristics contribute strongly to quality of life and ultimately impact access to and retention in care [1–3]. Specifically, sexual and gender minorities (SGM; i.e., men who have sex with men (MSM), transgender women (TGW), and non-binary individuals) living with HIV are at higher risk of reporting depression and other mental health symptoms compared to their cisgender counterparts not living with HIV [3–6]. Among several other factors, pervasive stigma and related discrimination that SGM living with HIV experience may be contributory factors [7–9]. Access to HIV-related and mental health services in Nigeria was hindered after the 2014 passage of the Same Sex Marriage Prohibition Act which further criminalized same-sex activities beyond the existing barriers in the Nigerian Criminal Code [10]. Criminalization at the federal level has exposed SGM throughout Nigeria to arrest, assault, harassment, and extortion at the hands of law enforcement and anti-LGBTQ vigilante groups [11,12]. Living under constant threat of violent and discriminatory acts, and without legal recourse in such cases, has left SGM in Nigeria at higher risk of experiencing depression and barriers to accessing HIV-related and mental health services [13,14].

The analyses below focus on Nigeria, but the research that motivates it comes from around the world. For example, based on related global studies [15,16], Scheim et al. in their meta-analyses reported that depression is associated with increased HIV risk behaviors among sexual minorities [17]. Young Black gay men living with HIV in the United States face high rates of depression and comorbidities, exacerbated by HIV stigma [18]. Similarly, in India, MSM and transgender women experience co-occurring psychosocial health issues, increasing sexual risk behaviors [19]. In sub-Saharan Africa, there are documented high depression rates (16–57%) among transgender women and cisgender MSM, with stigma contributing to this burden, particularly among transgender women [20].

However, there is a dearth of research exploring associations between HIV status, SGM identity, and depression symptomatology in Nigeria. This research is necessary to provide much-needed evidence for improving access to care for these vulnerable populations. Thus, we investigated the associations between HIV status, gender identity, and major depression symptomatology using cross-sectional analyses of baseline data collected as part of a prospective cohort study among SGM in Abuja, Nigeria.

## Methods

### Ethics statement

This study was approved by the institutional review boards of the University of Maryland Baltimore, Maryland, USA and the National and Federal Capital Territory Health Research Ethics Committees, Abuja, Nigeria. All participants provided written informed consent before enrollment.

### Study setting and procedures

This was a cross-sectional study of baseline data collected from July 2023 to December 2024 as part of a prospective cohort study examining non-communicable disease outcomes among SGM in Abuja, Nigeria (5R01HL165686–02) [21]. Participants were screened for eligibility and after obtaining their written informed consent, they were enrolled in the study. Recruitment, screening, and data collection occurred at an SGM-friendly special treatment clinic (TRUST) in Abuja. This clinic, which has existed since 2013, is managed by trained, culturally competent health care providers. It is supported by the International Center for Advocacy on Rights to Health —a community-based organization in Abuja that conducts advocacy programs, research, and human rights activism; the Institute of Human Virology Nigeria through funding from the United States President’s Emergency Plan for AIDS Relief (PEPFAR) program, the University of Maryland School of Medicine, and the National Institutes of Health (NIH) [22].

**Fig 1:**
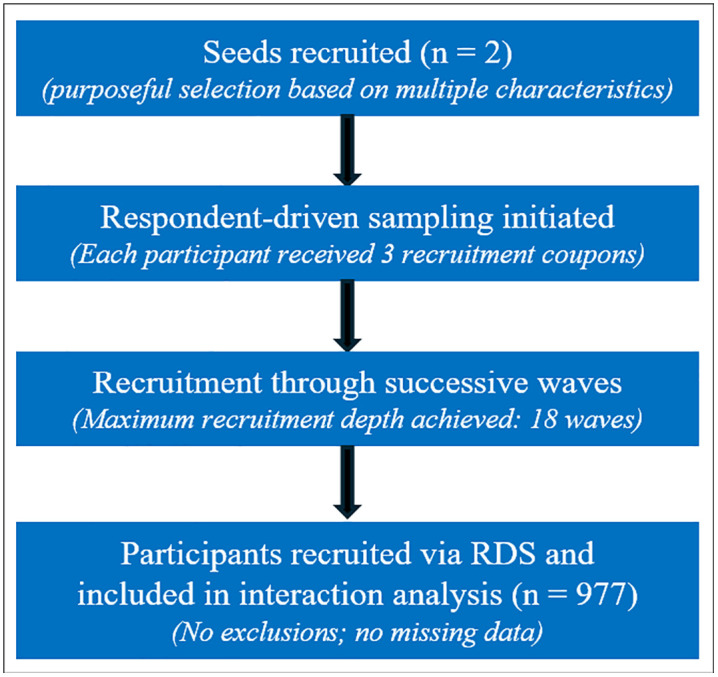
Participant recruitment using respondent-driven sampling.

Study participants were recruited using respondent-driven sampling (RDS), a method that has proven effective in obtaining representative samples of the hard-to-reach SGM population in Nigeria [23] and employed in previous studies for recruitment at the TRUST clinic and described in detail elsewhere [22,24]. In brief, an initial group of SGM called “seeds” are given study coupons to distribute to their SGM peers who may be interested in participating in the study as shown in Fig 1. Prospective participants who presented at the facility with valid coupons were recruited if they completed and met the eligibility screening criteria: individuals aged 18 years and above, assigned male at birth, had insertive or receptive anal sex in the past 12 months, and able and willing to provide informed consent. Participants completed a socio-behavioral questionnaire including questions about demographics and sexual behaviors, a depression assessment using the standardized Patient Health Questionnaire (PHQ-9) [25], had their HIV status confirmed using laboratory testing and medical records and provided anthropometric measurements. For this cross-sectional analysis, the sample comprised all participants enrolled in the ongoing prospective study by the time of analysis who had complete data on the exposure, outcome, and covariates of interest.

### Measures

Depression, the primary outcome for this study was measured using the PHQ-9, a commonly used scale with total scores of 0–27 [26]. Participants with scores below 10 were categorized as ‘non-depressed’, and those with scores of 10 and above were categorized as having ‘major depression symptomatology’ in line with standard clinical recommendations [13,27,28].

Our analyses incorporated a set of socio-demographic, behavioral, and clinical variables included a priori as covariates based on existing literature and clinical relevance given their potential to confound the association between gender identity, HIV status, and major depression symptomology. Demographic characteristics included age in years, level of education, employment, and marital status (many of the men in the sample are bisexual, married to women because of the strong social pressure in Nigeria for marriage and childbearing). Financial insecurity was defined as having difficulty paying for any of the following within the past three months: food, clothing, housing, medicine, health care, transportation, school fees, electricity, or phone subscription. Body mass index (BMI) was measured during clinical assessment and categorized as underweight (< 18.5 kg/m^2^), normal (18.5 kg/m^2^ to 24.9 kg/m^2^), or overweight (> 25.0 kg/m^2^) [29]. Current cigarette smoking and alcohol consumption were assessed via self-reports. Physical exercise was defined as engagement in work-related or recreational physical activity at least one day per week. Network size, defined as the number of other SGM personally known by the participant, was captured during the interview as part of the RDS protocol, and characterized by median and interquartile range (IQR) in analyses. Equilibrium was assessed using standard RDS diagnostics, and the network size information was used to evaluate RDS weighting.

### Statistical analyses

Descriptive characteristics were summarized using frequencies and proportions for categorical variables. Continuous variables were summarized by the median and IQR due to skewed distributions. Bivariable analyses evaluated associations between depression and each independent variable. Associations between depression and continuous variables were calculated using the Wilcoxon rank-sum test. Pearson’s chi-square test was used to evaluate bivariate associations between depression and categorical variables. In cases where expected cell counts were less than five in at least 20% of observations, Fisher’s exact test was used.

In our multivariable analyses, independent variables were treated as covariates. Candidate covariates were identified based on existing literature, and contextual relevance to depression, gender identity, and HIV status, and were further evaluated empirically for associations with both depression symptomatology and gender identity using Wilcoxon rank-sum, Pearson’s chi-square, or Fisher’s exact tests. A value of p ≤ 0.10 was used as a threshold to retain covariates with modest but potentially meaningful associations with depression and gender identity [30]. Gender identity was treated as the primary exposure of interest, while HIV status was included in all models as a key effect modifier to evaluate interaction with gender identity. Age was included in all models regardless of statistical significance. All independent variables were assessed for multicollinearity using variance inflation factors (VIFs) derived from a linear regression model that included the same set of predictors as the final logistic regression. A VIF value greater than 10 was considered indicative of multicollinearity. No evidence of multicollinearity was found among predictors in the multivariable model (all VIFs were below two). RDS equilibrium was reached within early recruitment waves, and differences between weighted and unweighted estimates were negligible; therefore, unweighted regression models are reported.

To examine the joint association of gender identity and HIV with depression, we fit a logistic regression model with the following independent variables: gender identity (transgender woman, non-binary, and cisgender man as reference), HIV status (PLHIV with PWoH as reference), their interaction terms and adjustment covariates for the gender-depression association (employment, financial insecurity, sexual partners and age). Employment status and financial insecurity were included as separate covariates to capture distinct socioeconomic dimensions, namely workplace exposure within a non-affirming social context and material deprivation, respectively. Odds ratios (OR) for joint associations (gender identity-cum-HIV) were generated with PWoH-cisgender man as the reference category. These were used to estimate the relative excess risk due to interaction (RERI) determined as: RERI = OR_11_ - OR_10_ - OR_01_ + OR_00_. RERI greater than zero indicates positive additive interaction (i.e., a supra-additive interaction), which suggests that the strength of the joint association is greater than the sum of individual association measures. All data collection and management were performed using the Research Electronic Data Capture (REDCap) application [31,32] and statistical analyses were performed using STATA 18 [33]. There were no missing data for variables included in the analyses; therefore, all enrolled participants were included in complete-case analyses.

## Results

### Socio-demographic and clinical characteristics

The median age of a total of 977 participants in this study was 28 years (IQR: 24 – 32). Of these, 43% (n = 422) had above senior secondary school education, 62% (n = 612) were employed, 48% (n = 472) reported at least one measure of financial insecurity, 5% (52) identified as queer, while 6% (n = 63) and 10% (n = 103) identified as transgender women and gender non-binary, respectively. HIV prevalence was high, with 55% (n = 543) of participants living with HIV, and the overall prevalence of major depression symptomatology was 18% (n = 175). High prevalence of major depression symptomatology was observed among participants who were underweight (29%), financially insecure (29%), or non-binary (26%) (Table 1). Compared to their referent subgroups, prevalence of major depression symptomatology was higher (p ≤ 0.05) among PLHIV (20.3% vs 15.0%); employed participants (20.1% vs 14.2%), those who were financially insecure (29.4% vs 7.1%), those who had only one sexual partner (22.2% vs 14.3%), and those who were underweight (29.2% vs 16.0%).

**Table 1:** Demographic, clinical and socio-behavioral characteristics by major depression symptomology among SGM in Abuja, Nigeria (n = 977).

Characteristics	All, n [%]	No major depression symptoms n (%)	Major depression symptomatology n (%)	p-value
	977 [100]	802 (82.1)	175 (17.9)	
Gender identity				**0.013** ^**C**^
Cisgender man	811 [83.0]	679 (83.7)	132 (16.3)	
Transgender woman	63 [6.4]	47 (74.6)	16 (25.4)	
Non-binary	103 [10.5]	76 (73.8)	27 (26.2)	
HIV status				**0.032** ^**C**^
PWoH	434 [44.4]	369 (85.0)	65 (15.0)	
PLHIV	543 [55.6]	433 (79.7)	110 (20.3)	
				
Age, median {IQR}	28 {24–32}	28 {24–33}	27 {24–32}	0.110^W^
				
Education				0.160^C^
<= SSS	555 [56.8]	464 (83.6)	91 (16.4)	
> SSS	422 [43.2]	338 (80.1)	84 (19.9)	
Employment				**0.021** ^**C**^
Unemployed	365 [37.4]	313 (85.8)	52 (14.2)	
Employed	612 [62.6]	489 (79.9)	123 (20.1)	
Financial insecurity				**<0.001** ^**C**^
Secure	505 [51.7]	469 (92.9)	36 (7.1)	
Insecure	472 [48.3]	333 (70.6)	139 (29.4)	
Sexual orientation				0.980^F^
Homosexual	342 [35.0]	282 (82.5)	60 (17.5)	
Bisexual	583 [59.7]	477 (81.8)	106 (18.2)	
Queer	52 [5.3]	43 (82.7)	9 (17.3)	
Sexual partner				**0.001** ^**C**^
One partner	451 [46.2]	351 (77.8)	100 (22.2)	
Multiple partners	526 [53.8]	451 (85.7)	75 (14.3)	
Marital status				0.570^F^
Never married	866 [88.6]	713 (82.3)	153 (17.7)	
Currently married	96 [9.8]	78 (81.3)	18 (18.8)	
Previously married	15 [1.5]	11 (73.3)	4 (26.7)	
Network size, median {IQR}	45 {30–60}	45 {30–60}	50 {30–60}	0.720^W^
				
Body mass index				**0.002** ^**C**^
Normal weight	687 [70.3]	577 (84.0)	110 (16.0)	
Underweight	120 [12.3]	85 (70.8)	35 (29.2)	
Overweight	170 [17.4]	140 (82.4)	30 (17.6)	
Physical exercise				0.360^C^
No	466 [47.7]	388 (83.3)	78 (16.7)	
Yes	511 [52.3]	414 (81.0)	97 (19.0)	
Smoking - current				0.058^C^
No	655 [67.0]	527 (80.5)	128 (19.5)	
Yes	322 [33.0]	275 (85.4)	47 (14.6)	
Alcohol use - current				0.074^C^
No	484 [49.5]	408 (84.3)	76 (15.7)	
Yes	493 [50.5]	394 (79.9)	99 (20.1)	

*Major depression symptomatology = PHQ-9 score >= 10; Non-depressed = PHQ-9 score <10; Acronyms: PHQ-9 = Patient Health Questionnaire; n[%] number and column percentage; n(%) number and row percentage; n{IQR} Median and interquartile range;* ^*W*^ *Wilcoxon rank-sum test;* ^*F*^ *Fisher's exact test,* ^*C*^ *Chi-square test, n Number of participants, IQR Interquartile range, SD Standard deviation, SGM Sexual and gender minority, HIV Human immunodeficiency virus, PLHIV Persons living with HIV, PWoH Persons without HIV, SSS senior secondary school, Physical exercise - engagement in physical exercise at least one day per week.*

### Prevalence and characteristics associated with depression

**Fig 2:**
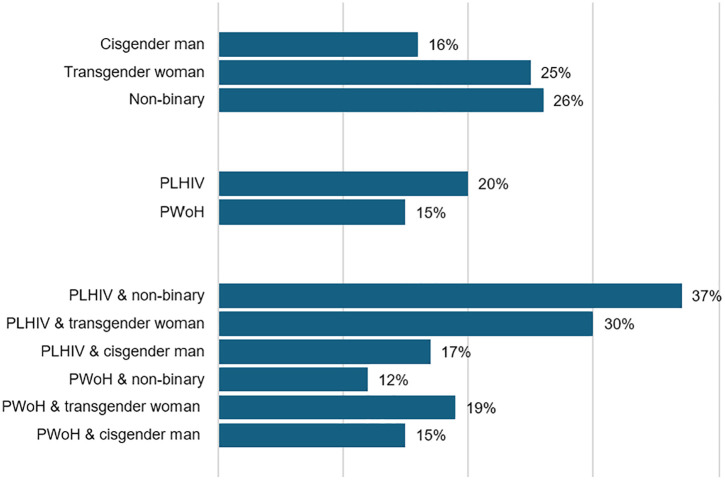
Prevalence of major depression symptomology by exposure and moderator included in regression analysis (n = 977).

Fig 2 shows prevalence of major depression symptomatology across joint categories of HIV status and gender identity: non-binary PLHIV had the highest percentage with major depression symptomatology (37%), followed by transgender women living with HIV (30%), while the lowest level of depression symptomatology was 12% among non-binary PWoH. In the univariable logistic regression, PLHIV had higher odds of major depression symptomatology compared to PWoH (OR = 1.44; 95% CI: 1.03–2.02); however, the estimated odds ratio was closer to the null, with confidence intervals including one in the multivariable logistic regression model (Table 2). In the multivariable model, the odds of major depression symptomatology were higher among participants who identified as transgender women (aOR 2.05; 95% CI: 1.09 – 3.88) or gender non-binary (aOR 2.38; 95% CI: 1.43 – 3.95) compared to cisgender men.

**Table 2:** Logistic regression for the association of characteristics with major depression symptomatology among sexual and gender minorities in Abuja, Nigeria (n = 977).

Major depression symptomatology	Univariable				Multivariable			
	OR	95%CI		P-value	OR	95%CI		P-value
***Exposure and moderator***								
Gender identity, (cisgender man)	ref				ref			
Transgender woman	1.75	0.96	3.18	0.066	2.05	1.09	3.88	**0.026**
Non-binary	1.83	1.13	2.94	0.013	2.38	1.43	3.95	**0.001**
HIV status, (PWoH)	ref				ref			
PLHIV	1.44	1.03	2.02	0.033	1.34	0.93	1.93	0.121

Acronyms: *OR – Odds ratio; CI – Confidence interval; PLHIV – Persons living with HIV; PWoH – Persons without HIV; ref – Reference group. Multivariable models adjust for age, employment status, financial insecurity, and number of sexual partners.*

### Joint associations of gender identity and HIV with depression

Table 3 shows measures for joint associations of gender identity and HIV status with depression symptomatology. The odds of depression symptomatology were higher among those identifying as transgender women living with HIV compared to cisgender men without HIV (aOR 2.50 [95% CI: 1.12 – 5.53]; RERI = 0.46). Similarly, the odds of depression symptomatology were higher among individuals identifying as non-binary and living with HIV compared to cisgender men without HIV (aOR 4.1 [95% CI: 2.16, 7.77]; RERI = 2.96). Both RERI estimates provide evidence of significant statistical interaction on the additive scale, indicating that with both HIV and non-cisgender identity had greater odds of depression symptomatology when compared to individuals with neither. We conducted a sensitivity analysis using a more stringent definition of depression, defined as severe depression symptomatology (PHQ-9 ≥ 15). In this analysis (Table 4), only the joint effect of being PLHIV and non-binary remained notable, with an odds ratio of 3.88 (95% CI: 1.95–7.72) and a positive additive interaction (RERI = 2.52), consistent with findings from the primary model. Other joint subgroups did not demonstrate significant associations under this definition. For the covariates in the model, the odds of major depression symptomology were also higher among participants who were employed (aOR 1.73; 95% CI: 1.18 – 2.52) or financially insecure (aOR 5.78; 95% CI: 3.77 – 8.86). Participants who reported multiple sexual partners had lowerr odds of major depression symptomology (aOR 0.61; 95% CI: 0.43 – 0.87).

**Table 3:** Joint associations of gender identity and HIV status with major depression symptomatology among sexual and gender minorities in Abuja, Nigeria (n = 977).

Major depression symptomatology			Multivariable ^A^			
Effect modification	n	RERI	OR	95%CI		p-value
PWoH & cisgender man ^I^	365		ref			
PWoH & transgender woman ^I^	26		1.90	0.69	5.24	0.214
PWoH & non-binary ^I^	43		1.00	0.37	2.65	0.995
PLHIV & cisgender man ^I^	446		1.14	0.75	1.72	0.542
PLHIV & transgender woman ^I^	37	0.46	2.50	1.12	5.53	**0.026**
PLHIV & non-binary ^I^	60	2.96	4.10	2.16	7.77	**<0.001**
Employment, (unemployed)			ref			
Employed			1.73	1.18	2.52	**0.005**
Financial insecurity, (secure)			ref			
Insecure			5.78	3.77	8.86	**<0.001**
Sexual partner, (one partner)			ref			
Multiple partners			0.61	0.43	0.87	**0.006**
Age in years			0.99	0.97	1.02	0.645

Acronyms: *I – Levels of interaction of HIV and Gender Identity; RERI – Relative excess risk due to interaction; OR – Odds ratio; CI – Confidence interval; PLHIV – Persons living with HIV; PWoH – Persons living without HIV; n = number of participants for each joint exposure category.*

**Table 4:** Sensitivity analysis: joint associations of HIV status and gender identity with severe depression symptomatology (PHQ-9 ≥ 15) among sexual and gender minorities in Abuja, Nigeria (n = 977).

Severe depression symptomatology (PHQ-9 ≥ 15)			Multivariable^A^			
Effect modification	n	RERI	OR	95%CI		p-value
PWoH & cisgender man^I^	365		ref			
PWoH & transgender woman^I^	26		2.57	0.93	7.09	0.068
PWoH & non-binary^I^	43		1.40	0.52	3.76	0.503
PLHIV & cisgender man^I^	446		0.96	0.60	1.53	0.855
PLHIV & transgender woman^I^	37		1.75	0.71	4.39	0.227
PLHIV & non-binary^I^	60	2.52	3.88	1.95	7.72	**<0.001**

A*cronyms: I – Levels of interaction of HIV and Gender Identity; RERI – Relative excess risk due to interaction; OR – Odds ratio; CI – Confidence interval; PLHIV – Persons living with HIV; PWoH – Persons living without HIV; n = number of participants for each joint exposure category. Model was adjusted for age, employment, financial insecurity, and sexual partnership as covariates included in the primary analysis.*

## Discussion

The overall prevalence of major depression symptomatology was 18% in this cohort of SGM, although the levels differed within subgroups defined by gender identity, HIV status, and other characteristics. Furthermore, we found strong evidence indicating that persons living with HIV and identifying either as transgender women or non-binary had an amplified likelihood of having major depression symptomatology beyond what was expected from the sum of individual risks.

These cross-sectional analyses provide evidence of the association between HIV status, gender identity, and depression symptomatology. Our findings highlight the importance of considering multiple individual characteristics when assessing SGM mental health, as overlooking these factors may lead to missing critical aspects of this population’s experiences. An HIV diagnosis signifies lifelong engagement in the healthcare system, which can pose unique challenges for SGM who do not identify as cisgender. For instance, transgender women may be compelled to present inauthentically to avoid harassment on the way to routine appointments or discrimination from medical staff. These specific challenges underscore the necessity of SGM-friendly clinics that are inclusive and welcoming to people of all gender identities and sexual orientations to promote long-term engagement and sustained quality of life. Our results align with prior research, which demonstrated that layered stigma exacerbates mental health disparities among SGM living with HIV, particularly in settings where gender identities and HIV status are heavily stigmatized [34–39]. Financial insecurity was strongly associated with major depression symptomatology [40], likely compounded by employment discrimination and limited financial opportunities [41–43].

In contrast to other studies, major depression symptomatology was more common among participants who were employed [44,45] The sociocultural context in Nigeria may play a role in this finding. It is possible that work environments hostile to SGM contribute to worsened mental health outcomes, with experiences of workplace discrimination, harassment, and social isolation potentially exacerbating depression symptomatology. This interpretation is corroborated by prior research indicating that SGM working in non-affirming environments experience higher levels of psychological distress [46–49]. It is important to note that employment status and financial insecurity, although conceptually related, did not exhibit collinearity in the model (VIF < 2), indicating they independently contributed to the risk of major depression symptomatology.

Participants with multiple sexual partners in our study had lower odds of exhibiting major depression symptomatology compared to those with only one partner. While research among females often links multiple sexual partnerships with increased psychosocial stress or risky health behaviors, multiple partnerships may affect outcomes differently among SGM populations in Nigeria [50,51]. In a hostile sociopolitical environment where sexual and gender minority identities are criminalized, having multiple partners may serve as a form of agency, autonomy, or social affirmation. Multiple partnerships may potentially reflect stronger integration within supportive networks or communities, which in turn may play a protective role in mental health. As other studies have demonstrated, strong peer connections and social capital among SGM individuals can enhance resilience and mental well-being [52,53]^.^ In contrast, individuals who feel more psychologically secure and less burdened by internalized stigma may be more likely to seek or maintain multiple partnerships. Future qualitative studies should explore how relationship dynamics intersect with mental health among SGM communities in Nigeria, uncovering nuanced meanings and psychosocial contexts.

Although this study provides a good starting point for future studies, it has some limitations. One limitation is the potential for misclassification of depression based on PHQ-9 scores. Another is self-reporting depression, employment, financial insecurity, and number of sex partners. Given the cross-sectional nature of this study, temporal or causal relationships between gender identity, HIV status, and depression symptomatology cannot be established.

The generalizability of the results of this study is limited to only men who have sex with men, transgender women, and non-binary persons. Transmasculine persons including transgender men assigned female at birth were excluded from the data collection due to overall considerations of the parent study (which is focused on populations at particularly high risk of HIV) and therefore could not be included in the analyses. We acknowledge the relatively small sizes of some gender identity subgroups in our study. However, diagnostic checks did not indicate undue influence of individual observations, and the effect sizes were consistent across models. Lastly, the findings of this study may not be generalizable to SGM living in other parts of Nigeria.

Future research should be informed by longitudinal data to make causal inferences and determine the directionality of relationships between depression, gender identity, and HIV status.

## Conclusion

As Nigeria continues working toward achieving epidemic control and an HIV-free future, health efforts need to shift focus from surviving to thriving. Such efforts for the healthy future of all Nigerians are consistent with the vision of the Lancet Nigeria Commission’s aims of investing in the health and the future of the nation [54]. This study is an important starting point for a better understanding of the mental health of SGM living with HIV, one of Nigeria’s most vulnerable populations. Building on other established risk factors, gender identity, financial insecurity, employment, social support networks and their interactive effects on mental health, the findings of this study should be investigated further to understand the mechanisms explaining these relationships and potential programmatic or policy interventions to improve SGM mental health through integration into existing HIV service delivery.
